# Diiodido[*N*′-(2-methoxy­benzyl­idene)-*N*,*N*-dimethyl­ethane-1,2-diamine]zinc(II)

**DOI:** 10.1107/S1600536809037209

**Published:** 2009-09-19

**Authors:** Xue-Wen Zhu, Zhi-Gang Yin, Chun-Xia Zhang, Xu-Zhao Yang, Gang-Sen Li

**Affiliations:** a Key Laboratory of Surface and Interface Science of Henan, School of Materials and Chemical Engineering, Zhengzhou University of Light Industry, Zhengzhou 450002, People’s Republic of China

## Abstract

In the title complex, [Zn(C_12_H_18_N_2_O)I_2_], the Zn^II^ ion is four-coordinated by the imine N and amine N atoms of the Schiff base ligand and by two iodide ions in a distorted tetra­hedral coordination.

## Related literature

For background to the chemistry of Schiff base complexes, see: Ali *et al.* (2008 ▶); Biswas *et al.* (2008 ▶); Chen *et al.* (2008 ▶); Darensbourg & Frantz (2007 ▶); Habibi *et al.* (2007 ▶); Kawamoto *et al.* (2008 ▶); Lipscomb & Sträter (1996 ▶); Tomat *et al.* (2007 ▶); Wu *et al.* (2008 ▶); Yuan *et al.* (2007 ▶). For related structures, see: Zhu (2008 ▶); Zhu & Yang (2008*a* ▶,*b* ▶,*c* ▶); Qiu (2006*a* ▶,*b* ▶); Wei *et al.* (2007 ▶); Zhu *et al.* (2007 ▶).
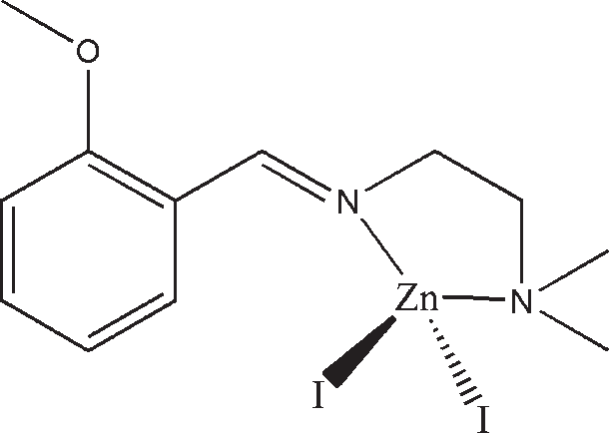


## Experimental

### 

#### Crystal data

[Zn(C_12_H_18_N_2_O)I_2_]
*M* *_r_* = 525.45
Monoclinic, 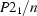

*a* = 13.5215 (8) Å
*b* = 7.2806 (4) Å
*c* = 18.4224 (11) Å
β = 109.250 (3)°
*V* = 1712.19 (17) Å^3^
*Z* = 4
Mo *K*α radiation
μ = 5.03 mm^−1^
*T* = 298 K
0.30 × 0.27 × 0.27 mm

#### Data collection

Bruker APEXII CCD area-detector diffractometer
Absorption correction: multi-scan (*SADABS*; Sheldrick, 2004 ▶) *T*_min_ = 0.314, *T*_max_ = 0.344
10129 measured reflections
3724 independent reflections
3128 reflections with *I* > 2σ(*I*)
*R*_int_ = 0.022

#### Refinement

*R*[*F* ^2^ > 2σ(*F* ^2^)] = 0.027
*wR*(*F* ^2^) = 0.069
*S* = 1.04
3724 reflections
166 parameters
H-atom parameters constrained
Δρ_max_ = 0.65 e Å^−3^
Δρ_min_ = −0.83 e Å^−3^

### 

Data collection: *APEX2* (Bruker, 2004 ▶); cell refinement: *SAINT* (Bruker, 2004 ▶); data reduction: *SAINT*; program(s) used to solve structure: *SHELXS97* (Sheldrick, 2008 ▶); program(s) used to refine structure: *SHELXL97* (Sheldrick, 2008 ▶); molecular graphics: *SHELXTL* (Sheldrick, 2008 ▶); software used to prepare material for publication: *SHELXL97*.

## Supplementary Material

Crystal structure: contains datablocks global, I. DOI: 10.1107/S1600536809037209/om2277sup1.cif

Structure factors: contains datablocks I. DOI: 10.1107/S1600536809037209/om2277Isup2.hkl

Additional supplementary materials:  crystallographic information; 3D view; checkCIF report

## Figures and Tables

**Table:** 

Zn1—N1	2.070 (3)
Zn1—N2	2.099 (3)
Zn1—I1	2.5538 (5)
Zn1—I2	2.5542 (4)

**Table:** 

N1—Zn1—N2	85.04 (11)
N1—Zn1—I1	105.44 (8)
N2—Zn1—I1	110.30 (8)
N1—Zn1—I2	121.86 (8)
N2—Zn1—I2	107.02 (8)
I1—Zn1—I2	121.063 (18)

**Fig. 1.**
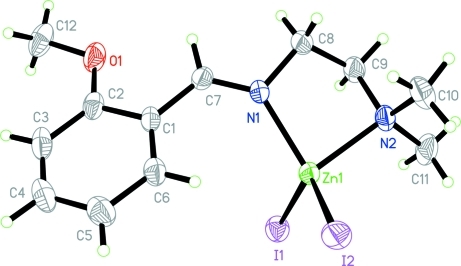
The molecular structure of the title complex, with ellipsoids drawn at the 30% probability level.

## supplementary crystallographic information

### Comment

Schiff bases have widely been used as versatile ligands in coordination chemistry (Biswas *et al.*, 2008; Wu *et al.*, 2008; Kawamoto *et al.*, 2008; Ali *et al.*, 2008; Habibi *et al.*, 2007), and their metal complexes are of great interest in many fields (Chen *et al.*, 2008; Yuan *et al.*, 2007; Tomat *et al.*, 2007; Darensbourg & Frantz, 2007). Zinc(II) is an important element in biological systems and functions as the active site of hydrolytic enzymes, such as carboxypeptidase and carbonic anhydrase where it is in a hard-donor coordination environment of nitrogen and oxygen ligands (Lipscomb & Sträter, 1996). Recently, we have reported a few Schiff base zinc complexes (Zhu, 2008; Zhu & Yang, 2008a,b,c). In this paper, the title new zinc(II) complex, Fig. 1, is reported.

In the title complex, the Zn^II^ atom is four-coordinated by the imine N and amine N atoms of the Schiff base ligand, and by two iodide ions in a tetrahedral coordination. The coordinate bond lengths (Table 1) are typical and comparable to the corresponding values observed in the Schiff base zinc complexes we reported previously and other similar Schiff base zinc complexes (Zhu *et al.*, 2007; Wei *et al.*, 2007; Qiu, 2006*a*,b).

### Experimental

The Schiff base compound was prepared by the condensation of equimolar amounts of 2-methoxybenzaldehyde with N,N-dimethylethane-1,2-diamine in a methanol solution. The complex was prepared by the following method. To an anhydrous methanol solution (5 ml) of ZnI_2_ (31.9 mg, 0.1 mmol) was added a methanol solution (10 ml) of the Schiff base compound (20.6 mg, 0.1 mmol) with stirring. The mixture was stirred for 30 min at room temperature and filtered. Upon keeping the filtrate in air for a few days, colorless block-shaped crystals were formed.

### Refinement

H atoms were placed in geometrically idealized positions and constrained to ride on their parent atoms, with C—H distances in the range 0.93–0.97 Å, and with *U*_iso_(H) = 1.2*U*_eq_(C).

### Crystal data

**Table:**

[ZnI_2_(C_12_H_18_N_2_O)]	*F*(000) = 992
*M**_r_* = 525.45	*D*_x_ = 2.038 Mg m^−^^3^
Monoclinic, *P*2_1_/*n*	Mo *K*α radiation, λ = 0.71073 Å
Hall symbol: -P 2yn	Cell parameters from 4576 reflections
*a* = 13.5215 (8) Å	θ = 2.3–27.0°
*b* = 7.2806 (4) Å	µ = 5.03 mm^−^^1^
*c* = 18.4224 (11) Å	*T* = 298 K
β = 109.250 (3)°	Block, colourless
*V* = 1712.19 (17) Å^3^	0.30 × 0.27 × 0.27 mm
*Z* = 4	

### Data collection

**Table:**

Bruker APEXII CCD area-detector diffractometer	3724 independent reflections
Radiation source: fine-focus sealed tube	3128 reflections with *I* > 2σ(*I*)
graphite	*R*_int_ = 0.022
ω scans	θ_max_ = 27.0°, θ_min_ = 1.6°
Absorption correction: multi-scan (*SADABS*; Sheldrick, 2004)	*h* = −14→17
*T*_min_ = 0.314, *T*_max_ = 0.344	*k* = −9→8
10129 measured reflections	*l* = −23→21

### Refinement

**Table:**

Refinement on *F*^2^	Primary atom site location: structure-invariant direct methods
Least-squares matrix: full	Secondary atom site location: difference Fourier map
*R*[*F*^2^ > 2σ(*F*^2^)] = 0.027	Hydrogen site location: inferred from neighbouring sites
*wR*(*F*^2^) = 0.069	H-atom parameters constrained
*S* = 1.04	*w* = 1/[σ^2^(*F*_o_^2^) + (0.0327*P*)^2^ + 0.876*P*] where *P* = (*F*_o_^2^ + 2*F*_c_^2^)/3
3724 reflections	(Δ/σ)_max_ = 0.001
166 parameters	Δρ_max_ = 0.65 e Å^−^^3^
0 restraints	Δρ_min_ = −0.82 e Å^−^^3^

### Special details

**Table:**

Geometry. All esds (except the esd in the dihedral angle between two l.s. planes) are estimated using the full covariance matrix. The cell esds are taken into account individually in the estimation of esds in distances, angles and torsion angles; correlations between esds in cell parameters are only used when they are defined by crystal symmetry. An approximate (isotropic) treatment of cell esds is used for estimating esds involving l.s. planes.
Refinement. Refinement of F^2^ against ALL reflections. The weighted R-factor wR and goodness of fit S are based on F^2^, conventional R-factors R are based on F, with F set to zero for negative F^2^. The threshold expression of F^2^ > 2sigma(F^2^) is used only for calculating R-factors(gt) etc. and is not relevant to the choice of reflections for refinement. R-factors based on F^2^ are statistically about twice as large as those based on F, and R- factors based on ALL data will be even larger.

### Fractional atomic coordinates and isotropic or equivalent isotropic displacement parameters (Å^2^)

**Table:**

	*x*	*y*	*z*	*U*_iso_*/*U*_eq_	
Zn1	0.98491 (3)	0.05442 (5)	0.79559 (2)	0.04115 (11)	
I1	1.14682 (2)	−0.09494 (4)	0.778684 (15)	0.05864 (9)	
I2	0.84884 (2)	−0.13708 (4)	0.830527 (15)	0.05832 (9)	
N1	0.9421 (2)	0.2610 (4)	0.71398 (14)	0.0415 (6)	
N2	1.0290 (2)	0.2698 (4)	0.87555 (15)	0.0466 (7)	
O1	0.9175 (2)	0.2845 (4)	0.49776 (13)	0.0643 (8)	
C1	0.8782 (3)	0.1069 (5)	0.59019 (17)	0.0419 (8)	
C2	0.8811 (3)	0.1219 (6)	0.51478 (19)	0.0497 (9)	
C3	0.8492 (3)	−0.0254 (7)	0.4646 (2)	0.0626 (11)	
H3	0.8534	−0.0178	0.4153	0.075*	
C4	0.8115 (3)	−0.1815 (7)	0.4873 (2)	0.0676 (12)	
H4	0.7887	−0.2782	0.4529	0.081*	
C5	0.8068 (3)	−0.1974 (6)	0.5608 (3)	0.0666 (11)	
H5	0.7820	−0.3048	0.5760	0.080*	
C6	0.8392 (3)	−0.0529 (5)	0.6115 (2)	0.0509 (9)	
H6	0.8348	−0.0628	0.6606	0.061*	
C7	0.9110 (3)	0.2645 (5)	0.64089 (17)	0.0434 (8)	
H7	0.9091	0.3785	0.6177	0.052*	
C8	0.9669 (4)	0.4396 (5)	0.7536 (2)	0.0592 (10)	
H8A	0.9051	0.4880	0.7626	0.071*	
H8B	0.9881	0.5263	0.7215	0.071*	
C9	1.0538 (3)	0.4168 (5)	0.8290 (2)	0.0545 (9)	
H9A	1.1184	0.3870	0.8195	0.065*	
H9B	1.0643	0.5316	0.8573	0.065*	
C10	0.9436 (3)	0.3264 (6)	0.9035 (2)	0.0680 (12)	
H10A	0.9593	0.4446	0.9277	0.102*	
H10B	0.9363	0.2381	0.9401	0.102*	
H10C	0.8793	0.3334	0.8610	0.102*	
C11	1.1229 (3)	0.2261 (7)	0.9423 (2)	0.0701 (12)	
H11A	1.1783	0.1852	0.9245	0.105*	
H11B	1.1061	0.1309	0.9724	0.105*	
H11C	1.1450	0.3340	0.9734	0.105*	
C12	0.9178 (4)	0.3156 (8)	0.4208 (2)	0.0781 (14)	
H12A	0.9665	0.2333	0.4099	0.117*	
H12B	0.9382	0.4402	0.4160	0.117*	
H12C	0.8488	0.2943	0.3851	0.117*	

### Atomic displacement parameters (Å^2^)

**Table:**

	*U*^11^	*U*^22^	*U*^33^	*U*^12^	*U*^13^	*U*^23^
Zn1	0.0480 (2)	0.0388 (2)	0.0354 (2)	−0.00454 (16)	0.01205 (17)	−0.00228 (15)
I1	0.05712 (17)	0.06509 (18)	0.05364 (16)	0.00639 (12)	0.01816 (12)	−0.00237 (12)
I2	0.06738 (18)	0.05101 (16)	0.06430 (17)	−0.01186 (12)	0.03217 (14)	−0.00014 (11)
N1	0.0504 (16)	0.0408 (15)	0.0324 (13)	−0.0016 (12)	0.0126 (12)	−0.0047 (11)
N2	0.0544 (17)	0.0496 (17)	0.0362 (14)	−0.0097 (14)	0.0154 (13)	−0.0080 (13)
O1	0.0774 (19)	0.082 (2)	0.0369 (13)	0.0063 (16)	0.0234 (13)	0.0029 (13)
C1	0.0382 (17)	0.054 (2)	0.0310 (15)	0.0080 (15)	0.0081 (13)	−0.0052 (14)
C2	0.0391 (18)	0.072 (3)	0.0357 (18)	0.0114 (17)	0.0085 (15)	−0.0034 (17)
C3	0.052 (2)	0.092 (3)	0.0386 (19)	0.015 (2)	0.0077 (17)	−0.017 (2)
C4	0.054 (2)	0.079 (3)	0.060 (3)	0.005 (2)	0.0060 (19)	−0.032 (2)
C5	0.059 (3)	0.062 (3)	0.071 (3)	0.000 (2)	0.009 (2)	−0.015 (2)
C6	0.047 (2)	0.060 (2)	0.0409 (18)	−0.0006 (17)	0.0071 (15)	−0.0091 (17)
C7	0.0468 (19)	0.0472 (19)	0.0363 (17)	0.0048 (15)	0.0139 (14)	0.0033 (14)
C8	0.093 (3)	0.0391 (19)	0.0430 (19)	−0.0089 (19)	0.018 (2)	−0.0042 (16)
C9	0.073 (3)	0.047 (2)	0.0431 (19)	−0.0207 (18)	0.0192 (18)	−0.0067 (16)
C10	0.080 (3)	0.069 (3)	0.066 (3)	−0.009 (2)	0.040 (2)	−0.023 (2)
C11	0.075 (3)	0.086 (3)	0.0367 (19)	−0.013 (2)	0.0007 (19)	−0.003 (2)
C12	0.075 (3)	0.123 (4)	0.041 (2)	0.017 (3)	0.026 (2)	0.015 (2)

### Geometric parameters (Å, °)

**Table:**

Zn1—N1	2.070 (3)	C5—C6	1.378 (5)
Zn1—N2	2.099 (3)	C5—H5	0.9300
Zn1—I1	2.5538 (5)	C6—H6	0.9300
Zn1—I2	2.5542 (4)	C7—H7	0.9300
N1—C7	1.272 (4)	C8—C9	1.504 (5)
N1—C8	1.475 (4)	C8—H8A	0.9700
N2—C10	1.471 (5)	C8—H8B	0.9700
N2—C9	1.477 (4)	C9—H9A	0.9700
N2—C11	1.482 (5)	C9—H9B	0.9700
O1—C2	1.358 (5)	C10—H10A	0.9600
O1—C12	1.437 (4)	C10—H10B	0.9600
C1—C6	1.386 (5)	C10—H10C	0.9600
C1—C2	1.407 (5)	C11—H11A	0.9600
C1—C7	1.454 (5)	C11—H11B	0.9600
C2—C3	1.388 (6)	C11—H11C	0.9600
C3—C4	1.366 (6)	C12—H12A	0.9600
C3—H3	0.9300	C12—H12B	0.9600
C4—C5	1.381 (6)	C12—H12C	0.9600
C4—H4	0.9300		
			
N1—Zn1—N2	85.04 (11)	N1—C7—C1	126.1 (3)
N1—Zn1—I1	105.44 (8)	N1—C7—H7	117.0
N2—Zn1—I1	110.30 (8)	C1—C7—H7	117.0
N1—Zn1—I2	121.86 (8)	N1—C8—C9	109.9 (3)
N2—Zn1—I2	107.02 (8)	N1—C8—H8A	109.7
I1—Zn1—I2	121.063 (18)	C9—C8—H8A	109.7
C7—N1—C8	116.6 (3)	N1—C8—H8B	109.7
C7—N1—Zn1	134.4 (2)	C9—C8—H8B	109.7
C8—N1—Zn1	108.5 (2)	H8A—C8—H8B	108.2
C10—N2—C9	110.8 (3)	N2—C9—C8	111.0 (3)
C10—N2—C11	109.0 (3)	N2—C9—H9A	109.4
C9—N2—C11	110.0 (3)	C8—C9—H9A	109.4
C10—N2—Zn1	112.4 (2)	N2—C9—H9B	109.4
C9—N2—Zn1	101.56 (19)	C8—C9—H9B	109.4
C11—N2—Zn1	112.9 (3)	H9A—C9—H9B	108.0
C2—O1—C12	119.0 (4)	N2—C10—H10A	109.5
C6—C1—C2	118.8 (3)	N2—C10—H10B	109.5
C6—C1—C7	123.0 (3)	H10A—C10—H10B	109.5
C2—C1—C7	118.1 (3)	N2—C10—H10C	109.5
O1—C2—C3	125.2 (3)	H10A—C10—H10C	109.5
O1—C2—C1	115.2 (3)	H10B—C10—H10C	109.5
C3—C2—C1	119.6 (4)	N2—C11—H11A	109.5
C4—C3—C2	120.3 (4)	N2—C11—H11B	109.5
C4—C3—H3	119.9	H11A—C11—H11B	109.5
C2—C3—H3	119.9	N2—C11—H11C	109.5
C3—C4—C5	120.8 (4)	H11A—C11—H11C	109.5
C3—C4—H4	119.6	H11B—C11—H11C	109.5
C5—C4—H4	119.6	O1—C12—H12A	109.5
C6—C5—C4	119.5 (4)	O1—C12—H12B	109.5
C6—C5—H5	120.2	H12A—C12—H12B	109.5
C4—C5—H5	120.2	O1—C12—H12C	109.5
C5—C6—C1	121.0 (4)	H12A—C12—H12C	109.5
C5—C6—H6	119.5	H12B—C12—H12C	109.5
C1—C6—H6	119.5		
